# Role of exosomes and exosomal microRNA in muscle–Kidney crosstalk in chronic kidney disease

**DOI:** 10.3389/fcell.2022.951837

**Published:** 2022-09-07

**Authors:** Sijie Zhou, Gladys Lai Ying Cheing, Alex Kwok Kuen Cheung

**Affiliations:** ^1^ Department of Rehabilitation Sciences, The Hong Kong Polytechnic University, Hong Kong SAR, China; ^2^ Department of Nephrology, The First Affiliated Hospital of Zhengzhou University, Zhengzhou, China

**Keywords:** exosomes, microRNA, skeletal muscle, chronic kidney disease, crosstalk

## Abstract

Chronic kidney disease (CKD) is a progressive damage of kidneys that can no longer serve the blood-filtering function, and is a life-threatening condition. Skeletal muscle wasting is a common complication of CKD. Yet the relationship between kidney and skeletal muscle in CKD remains unclear. Exosomes, a type of small membrane-bound vesicles released from cells to the extracellular environment, have increasingly received attention due to their potential as mediators of crosstalk between kidneys and different organs, including skeletal muscle. This mini-review summarizes the recent findings that point to the role of exosomes in the cross-talk between kidney and skeletal muscle in CKD. Understanding of the contents and the mechanism of exosome release may prone exosomes be the potential therapeutic targets for CKD.

## Introduction

Extracellular vesicles (EVs) are small membrane-bound vesicles released from cells into extracellular environments (Sahoo et al., 2021) such as plasma, cerebrospinal fluid, urine, saliva, amniotic fluid, colostrum, breast milk, synovial fluid, semen, and pleural ascites (Song et al., 2020). Recently, EVs have been recognized as important players in cell-to-cell and inter-tissue communication and in maintaining homeostasis (Qin and Dallas, 2019; Díaz-Garrido et al., 2021). The three major types of EVs are exosomes (<100 nm), microvesicles (<1,000 nm), and apoptotic bodies (>1,000 nm), which are distinguishable by their size, biogenesis, release pathways, content, function, and expressed biomarkers (Akers et al., 2013; Doyle and Wang, 2019). Accumulating evidence suggests that cells can communicate with neighboring or distant cells, tissues, and organs through the exosomes (Kalluri and LeBleu, 2020).

Exosomes are released not only by healthy cells or organs but also by injured, stressed, and diseased cells or organs (Yuana et al., 2013). In recent years, the role of exosomes in organ crosstalk has been extensively studied in various disease models, including chronic kidney disease (CKD) models (Zhang et al., 2018; Wang et al., 2019a; Wang et al., 2020). CKD is a global public health concern and is prevalent in 10–15% of the adult population worldwide (Levin et al., 2017). CKD can eventually progress to kidney failure, also known as end-stage renal disease (ESRD). Patients with end-stage renal failure must receive dialysis or kidney transplantation for survival. In addition to progressive decline in renal function, CKD is commonly associated with multiple complications which contribute to high morbidity, adverse medical outcomes, and poor quality of life (Bello et al., 2011). One of the most frequent complications of CKD is skeletal muscle wasting, which is characterized by the loss of muscle mass, strength, and function (Tsai et al., 2017) and an increase in morbidity and mortality (Roshanravan et al., 2013). Other complications of CKD directly or indirectly associated with skeletal muscle wasting are an increased risk of insulin resistance (Carré and Affourtit, 2019), cardiometabolic disease (Harada et al., 2017), and mineral and bone disorders (Karava et al., 2020). Roshanravan et al. demonstrated that exercise ameliorates muscle impairment and improves physical function and performance, leading to clinically important benefits for kidneys with CKD (Roshanravan et al., 2017). Increasing muscle mass protects against the progression of several kidney diseases (Peng et al., 2017; Zhang et al., 2018; Wang et al., 2020), and ameliorating skeletal muscle atrophy has been shown to improve kidney recovery after injury by reducing renal fibrosis (Hanatani et al., 2014; Rondon-Berrios et al., 2014). There is potential crosstalk between skeletal muscle and kidney, and recent research suggests that exosomes are one of the mediators in such crosstalk. Understanding the features and roles of exosomes may shed light on the development of novel therapeutic strategies for CKD with muscle wasting.

In this review, we survey exosome biogenesis and summarize the current literature with regard to the functions of exosomes in muscles and kidneys and their role in mediating crosstalk between these two tissues. We also review the roles of exosomes in pathogenesis and discuss therapies for people with CKD-associated muscle wasting.

### Biogenesis, release, and uptake of exosomes

Exosomes contain almost 10,000 different proteins, over 1,000 different types of lipids, and approximately 3,000 each of coding and non-coding nucleic acids (Jeppesen et al., 2019; Skotland et al., 2019; Wang et al., 2021). Exosomal proteins include membrane transport and fusion-related proteins (e.g. annexin, Rab-GTPase, and HSPs) for exosome trafficking (Dai et al., 2020), tetraspanins (e.g. CD9, CD63, CD81, CD82, CD106, and Tspan8) for facilitating the entry of exosomal contents into exosomes (Dai et al., 2020), proteins related to multivesicular bodies (MVBs) (e.g. ALG-2-interacting protein X and tumor suppressor gene 101) for sorting cargo into exosomes (Willms et al., 2016), and cytoskeletal proteins (e.g. actin, tubulin, and myosin) (Dai et al., 2020). Exosomal lipids essential for maintaining exosome morphology and exosome biogenesis and regulating homeostasis in recipient cells include cholesterol, sphingomyelin, glycosphingolipids, phosphatidylserine, and ceramides (Skotland et al., 2017). Exosomal nucleic acids consist of mRNAs, microRNAs, lncRNAs, circRNAs, rRNAs, tRNAs, snoRNAs, snRNA, and piRNAs. Exosomes transfer the RNAs from parent cells to target cells or tissues and exert specific cellular functions (van den Boorn et al., 2013). Among various contents within exosomes, miRNA has been receiving extensive focuses due to the number of candidates and diverse functions (Table 1).

**TABLE 1:** Exosomal miRNAs in Muscle and/or Kidney

miRNAs	Model/Disease	Function	Reference
miR-145-5p	Bu-Cy treated mice	Maintain skeletal muscle mass	Cho et al. (2021)
miR-133b, miR-181a-5p	Acute excercise in humans	Muscle communication	Guescini et al. (2015)
miR-486-5p, miR-215-5p miR-941	Regular excercise in humans	Biomakers for excercise	Nair et al. (2020)
miR-151b	Regular excercise in humans	Biomakers for excercise	Nair et al. (2020)
miR-133a	HIIT in mice	Biomakers for excercise	Castaño et al. (2020)
miR-133b	HIIT in mice	Improving glucose tolerance and insulin sensitivity	Castaño et al. (2020)
miR-29c-3p	Ambulant DMD Patients	Novel noninvasive biomaker for Ambulant DMD	Catapano et al. (2018)
miR-23b-3p, miR-21-5p	Nonambulant DMD Patients	Novel noninvasive biomaker for Nonambulant DMD	Catapano et al. (2018)
miR-199a-5p	Max-mice	Including phenotypic conversion of normal fibroblasts to myofibroblasts	Zanotti et al. (2018)
MiR-1, miR-133a, miR-206	Max mice	Including phenotypic conversion of normal fibroblasts to myofibroblasts	Matsuzaka et al. (2016)
miR-182	STZ mice	Attenuating muscle atrophy by targeting FoxO3	Hudson et al. (2014)
miR-21	UUO mice	Accelerating the devleopment of renal fibrosis by activatiing fibroblasts	Zhao et al. (2021)
miR-25-3p	High Glucose induced podocytes	Enhancing podocyte survival by suppressing DUSP1	Huang et al. (2020)
miR-145, miR-130	Type 1 diabetic patients	Biomakers for microalbuminuric diabetic patients	Barutta et al. (2013)
miR-320c, miR-6068, miR-1234-5p, miR-6133, miR-4270, miR-4739, miR-371b-5p, miR-638, miR-572, miR-1227-5p, miR-6162, miR-1915-5p, miR-4778-5p, miR-2861	Type 2 diabetic nephropathy patients	Biomakers for Type 2 diabetic nephropathy patients	Delić et al. (2016)
miR-30d, miR-30e-5p	Type 2 diabetic netropathy patients	Biomakers for Type 2 diabetic nephropathy patients	Delić et al. (2016)
miR-374a-5p	UUO mice	Inhibiting the Progression of renal fibrosis by regulating MAPK6/MK5/YAP axis	Liang et al. (2022)
miR-186-5p	STZ rats	Attenuating renal fibrosis by downregulation of smad5	Yang et al. (2022)
miR-125a	STZ rats	Inhibiting Diabetic Nephropathy progression via inhibition of HDAC1 and ET-1	Hao et al. (2021)
miR-23a, miR-27a	STZ mice	Preventing diabetes-included muscle cachexia and attenuates renal fibrosis via regulating Akt, PTEN, and FoxO1	Zhang et al. (2018)
miR-23a, miR-27a	5/6 nephrectomy mice	Attenuating muscle loss, improving grip strength, increasing the phosphorylation of Akt and FoxO1, decreasing the activation of phosphat	Wang et al. (2017)
miR-26a	5/6 nephrectomy mice	Increasing the skeletal muscle cross-sectional area, decreasing the upregulation of the FBXO32/atrogin-1 and TRIM63/MuRF1 and Depressing cardiac fibrosis lesions	Wang et al. (2019b)
miR-26a	UUO mice	Preventing muscle atrophy by inhibiting the transcription factor FoxO1, Limiting renal fibrosis by suppressing CTGF	Zhang et al. (2019)
miR-26a	UUO mice	Ameliorating skeletal muscle atrophy and attenuating kidney fibrosis by downregulating YY1, TGF-B pathway and some fibrotic-related proteins	Wang et al. (2019a), Wang et al. (2020)

Abbrivation: Bu, busulfan; Cy, cyclophosphamide; HIIT, high-intensity interval training; DMD, duchenne muscular dystrophy; FoxO3, forkhead box protrin O3; DUSP1, dual specificity protein phosphatase 1; MAPK6, Mitogen-activated protein kinase 6; YAP, yes-associated protein; ET-1, endothelin-1; HDAC1, histone deacetylation 1; PTEN, phosphatase and tensin homolog; MuRF-1, muscle ring-finger protein-1; FoxO1, forkhead box protein O1; TRIM63, Tripartite Motif Containing 63; CTGF, connective tissue growth factor; YY1, Yin Yang 1.

**FIGURE 1:**
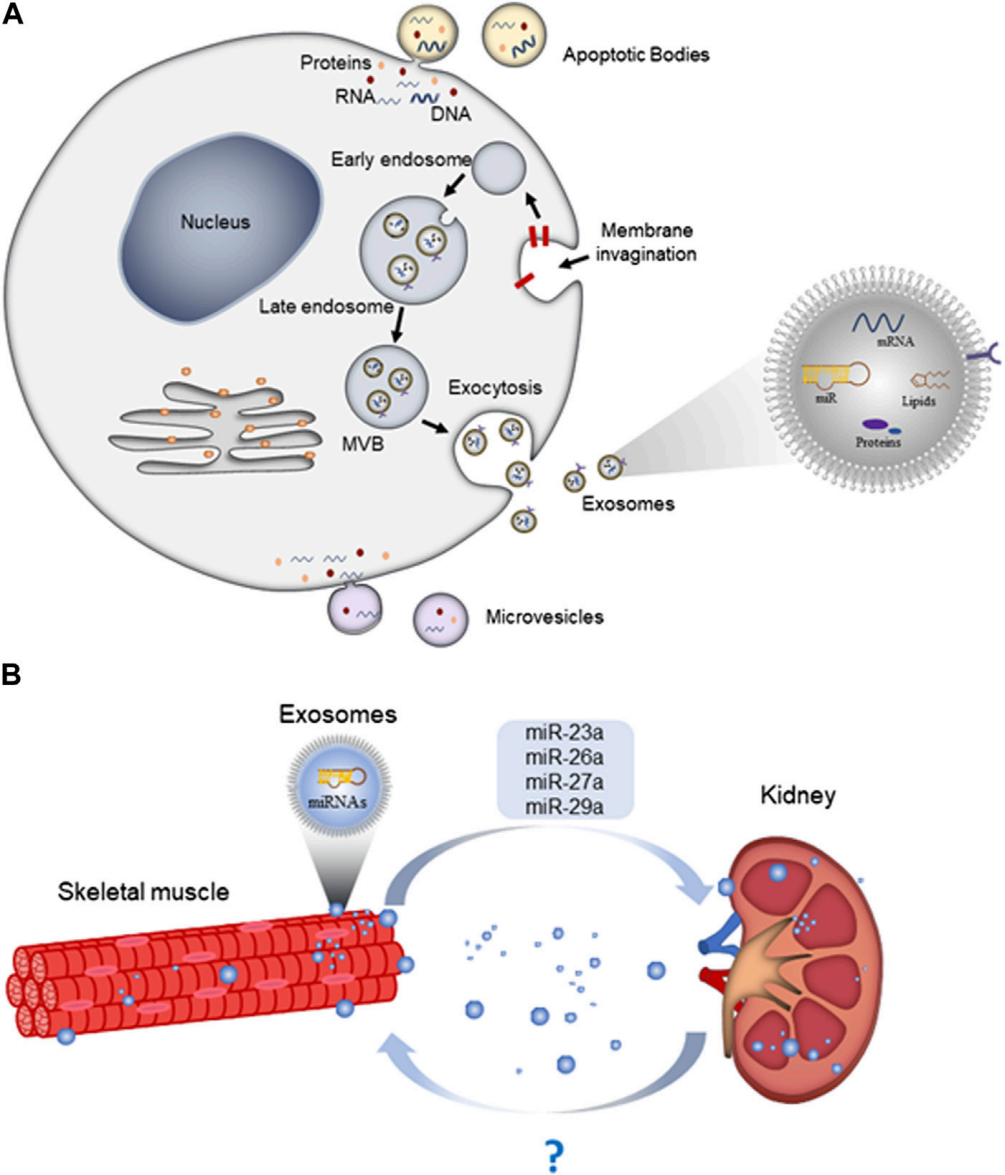
Exosome biogenesis, release, and as mediators for organ crosstalk. **(A)** Exosomes are formed by the endosomal system, including invagination of the endocytic membrane, early endosomes, late-endosomes, and multivesicular bodies (MVBs). Subsequently, large MVBs fuse with the cellular plasma membrane and release exosomes into the extracellular space; **(B)** skeletal muscle-derived exosomes can travel along the circulation and deliver miRNAs to kidney and exert specific functions.

Exosomes originate from inward budding and invagination of the plasma membrane that forms early endosomes. Early endosomes mature into late endosomes, which then undergo invagination to form intraluminal vesicles (ILV) within large MVBs (McAndrews and Kalluri, 2019). When MVBs mature and eventually merge with the plasma membrane, exosomes are released into the extracellular space (Williams and Urbé, 2007) (Figure 1A).

The release of exosomes depends on the activity of and interaction between cytoskeleton (microtubule and microfilament), Rab-GTPase, molecular motors (dynein and kinesin), and fusion apparatus SNARE (soluble N-ethylmaleimide-sensitive factor attachment protein receptors) complex (Hessvik and Llorente, 2018). Once secreted, exosomes are taken up by recipient cells through at least three different mechanisms: endocytosis, direct fusion with plasma membrane, or receptor-ligand interaction (Yue et al., 2020).

### Skeletal muscle exosomes in cell–cell communication

Skeletal muscle exosomes were first discovered in early 2010 by Guescini et al. using western blotting and transmission electron microscopy (Guescini et al., 2010). These exosomes contain special markers such as TSG101 and ALIX, which were visualized by immunogold labeling. Proteomic analysis of exosomes revealed that some signal transduction proteins, including guanine nucleotide-binding proteins, small GTP-binding proteins, and 14-3-3 proteins, are part of the exosome-associated proteins in skeletal muscle (Guescini et al., 2010). Several miRNAs that are only or preferentially expressed in skeletal muscle are called myomiRs (myo = muscle + miR = miRNA) (McCarthy, 2008). Studies have shown that when muscle cells are subjected to damage, the levels of miRNAs within muscle are reduced, and instead of being downregulated or passively leaked as previously believed, they are packaged into exosomes and transferred into circulation during specific periods of muscle regeneration (Coenen-Stass et al., 2016; Siracusa et al., 2016). These findings indicate that exosomal miRNAs are actively participating in muscle regeneration; in fact, myomiRs, including miR-1, miR-133a, miR-133b, miR-206, miR-208b, miR-486, and miR-499, have been shown to play significant roles in different stages of skeletal muscle development (Zilahi et al., 2019).

Exosomes can mediate cell–cell communication via transfer proteins, mRNAs, lncRNAs, circRNAs, or miRNAs directly to recipient cells. Muscle-derived exosomes play an important role in crosstalk between myoblasts and myotubes. Exosomes secreted from myotubes in well-differentiated C2C12 culture suppressed myoblast proliferation of myoblasts via downregulation of cyclin D1 and induced differentiation via upregulation of myogenin (Forterre et al., 2014). In well-differentiated C2C12 culture subjected to treatment of tumor necrosis factor-α and interferon-γ, the myotubes exhibited stress responses by upregulating adenosine monophosphate-activated protein kinase signaling pathways, which in turn triggered the release of exosomes containing atrophic signals such as myostatin and MAFbx and thus inhibited expression of myogenic regulatory factor MyoD and myogenin (Kim et al., 2018).

Muscle-derived exosomes can also travel along the circulation and deliver cargo to other cells and organs, where they exert particular functions on the target organs. Fluorescent-labeled muscle-derived exosomes injected through the tail vein of mice were found to be taken up into the lung, liver, spleen, brain, heart, pancreas, and GI tract within one day (Aswad et al., 2014). Skeletal-muscle-derived exosomes induces angiogenesis *via* ROS-activated nuclear factor-κB (NF-κB) signaling in cultured human umbilical vein endothelial cells (HUVECs) (Nie et al., 2019).

Skeletal muscle cells not only release exosomes but also uptake exosomes from other cells. Intramuscular injection of bone marrow stromal cell (BMSC) exosomes into mice after muscle contusion alleviated the inflammatory response, reduced fibrosis size, promoted muscle regeneration, and improved biomechanical properties through macrophage polarization (Luo et al., 2021). In another study, the authors showed that activin A induced Smad2/3 and FoxO1 nuclear translocation and transcriptional upregulation of Atrogin-1 and MuRF-1 genes, which resulted in muscle atrophy. Exosomal miR-145-5p released from tonsil-derived mesenchymal stem cells has been shown to maintain or improve skeletal muscle mass in various activin-A-elevated pathologic conditions (Cho et al., 2021).

### Muscle exosomes induced by exercise

Exercise imparts well-known benefits to multiple organs, including the muscles, kidneys, heart, lungs, and immune system (Landi et al., 2014; Brellenthin et al., 2019; Abd El-Kader and Al-Jiffri, 2020; Sanz-Santiago et al., 2020; Duan et al., 2021). A recent study showed that exercise can induce exosome production and miRNA processing in muscle (Garner et al., 2020). When released into the circulation, exercise-mediated skeletal-muscle exosomes (i.e. exersomes) containing exerkines (peptides, nucleic acids, lipids, and miRNA species) play an important role in crosstalk between skeletal muscle and distal organs (e.g. pancreas, liver, heart, brain, kidney, adipose tissue, and skin) (Safdar et al., 2016). The expression of circulating miRNA is altered in response to exercise (Banzet et al., 1985; Aoi et al., 2013; Sawada et al., 2013). Guescini et al. reported that the expression level of miR-181a-5p and miR-133b in muscle-derived circulating exosomes was elevated after acute exercise, and they found a positive correlation between aerobic fitness and muscle-specific miRNAs (Guescini et al., 2015). Nair et al. found that regular exercise significantly increased the baseline expression of exosomal miR-486-5p, miR-215-5p, and miR-941 and decreased expression of exosomal miR-151b (Nair et al., 2020). Interestingly, it was reported that miR-133a expression in skeletal muscle increased upon acute exercise but decreased after prolonged exercise training (Nielsen et al., 2010). On the other hand, Castaño et al. showed that high-intensity interval training (HIIT) significantly increased muscle-derived exosomal miR-133a and miR-133b in circulation (Castaño et al., 2020). Moreover, muscle-derived exosomal miR-133b improved glucose tolerance and insulin sensitivity and decreased plasma levels of triglycerides via suppressing FoxO1 expression and hepatocyte glucose production (Castaño et al., 2020).

Overall, accumulating evidence supports that exercise not only changes muscle-derived exosomes but also mediates the beneficial effects on other tissues via exosomal microRNAs.

### Exosomal miRNAs in muscular diseases

Emerging evidence suggests that muscular diseases can alter the cargo of muscle-derived exosomes. Catapano et al. reported that the level of exosomal miR-29c-3p in urine was significantly reduced in ambulant (A) Duchenne muscular dystrophy (DMD) patients, while the levels of exosomal miR-23b-3p and miR-21-5p in urine were significantly downregulated in nonambulant (NA) DMD patients compared with controls (Catapano et al., 2018). The study indicated that urinary exosomes miR-29c-3p, miR-23b-3p, and miR-21-5p were potential noninvasive diagnostic biomarkers for DMD. Zanotti et al. reported that exosomes released by muscle-derived fibroblasts of DMD patients had significantly higher levels of miR-199a-5p than control exosomes. Injecting DMD fibroblast-derived exosomes that contain elevated levels of miR-199a-5p can lead to excessive skeletal muscle fibrosis (Zanotti et al., 2018), and this study demonstrated that exosomes could mediate pathogenic effects in muscular diseases. In contrast, other studies demonstrated the protective roles of exosomes in muscular diseases. Matsuzaka et al. showed that C2C12 myoblast-derived exosomes are engineered to overexpress myomiR-1, myomiR-133a, and myomiR-206 that can improve survival of C2C12 myoblasts (Matsuzaka et al., 2016). Hudson et al. reported that dexamethasone increases the level of C2C12 myotube-derived exosomal miR-182, which can attenuate atrophy-related gene expression by targeting FoxO3 in skeletal muscle (Hudson et al., 2014). This research provides the basis for future applications of exosomes and exosomal miRNAs as a novel biological therapeutic approach for treating muscular diseases.

### Function of exosomal miRNAs in chronic kidney disease

Numerous recent studies have demonstrated that exosomal miRNAs participate in the pathogenesis of CKD. The roles of exosomal miRNAs in CKD have been widely studied, especially in renal fibrosis induced by unilateral ureteral obstruction (UUO) and in models of diabetic nephropathy (DN). For example, in TGF-β1-treated NRK-52E renal epithelial cells, fibrotic progression was associated with exosomal secretion (Zhao et al., 2021). These TGF-β1-induced exosomes were found to contain high levels of miR-21, and when isolated and injected into the obstructed kidneys, they activated fibroblasts and triggered renal fibrosis via the PTEN/Akt pathway (Zhao et al., 2021). Inhibition of miR-21 expression abolished the fibrotic progression, suggesting that miR-21 mediated the TGF-β1-induced renal fibrosis. In diabetic nephropathy, which is one of the common causes of CKD, podocyte injury is crucial for disease progression. Using a hyperglycemia-induced podocyte injury model *in vitro*, Huang et al. showed that podocyte injury was promoted when co-cultured with M1 macrophages but was ameliorated when co-cultured with M2 macrophages. Subsequently, the authors found that M2 macrophages expressed high levels of exosomal miR-25-3p, and it was this particularly exosomal miRNA that enhanced podocyte survival by suppressing expression of DUSP1, a known cell autophagy inhibitor (Huang et al., 2020). These studies demonstrate that exosomal miRNAs could be therapeutic targets for CKD.

Barutta et al. reported that miR-145 and miR-130a were enriched in urinary exosomes from type 1 diabetic patients with incipient diabetic nephropathy compared with type 1 diabetic patients without kidney damage. Moreover, the high glucose level induced a marked increase in the level of mesangial-cell-derived exosomal miR-145 levels (Barutta et al., 2013). Delić et al. showed increased levels of urinary exosomal miRNA, including miR-320c, miR-6068, miR-1234-5p, miR-6133, miR-4270, miR-4739, miR-371b-5p, miR-638, miR-572, miR-1227-5p, miR-6126, miR-1915-5p, miR-4778-5p, and miR-2861, but they found decreased miR-30d-5p and miR-30e-5p in type 2 diabetic nephropathy patients compared with healthy donors and type 2 diabetic patients without kidney damage (Delić et al., 2016). Regardless, the functions of individual exosomal miRNA, such as dynamic and differential expression, reflected the potential use of exosomal miRNAs as biomarkers for diagnostic purposes.

Several studies have shown that exosomal miRNAs derived from mesenchymal stem cells (MSCs) exhibit therapeutic benefits by suppressing kidney damage in CKD models. Liang et al. showed that exosomal miR-374a-5p derived from MSCs prevents the progression of renal fibrosis by regulating the MAPK6/MK5/YAP axis in renal fibrotic mice (Liang et al., 2022). Yang et al. reported that exosomal miR-186-5p derived from MSCs attenuated renal fibrosis *in vitro* and *in vivo* by downregulation of Smad5 (Yang et al., 2022). Hao et al. suggested that MSC-derived exosomal miR-125a inhibits DN progression and alleviates the symptoms *via* inhibition of histone deacetylase 1 and endothelin-1 in streptozotocin-treated rats and high-glucose-treated glomerular mesangial cells (Hao et al., 2021). Taken together, these findings indicate the potential roles of exosomal miRNAs for therapeutic intervention in CKD models.

### Role of exosomal miRNAs in muscle–kidney crosstalk

Skeletal muscle wasting is one of the most common complications of CKD. It is believed that catabolic/anabolic imbalance is a major contributive factor to skeletal muscle wasting (Robinson et al., 2020). The IGF-1-Akt-mTOR pathway is a key promotor to muscle growth. In CKD, metabolic acidosis, chronic inflammatory responses, increased elevated glucocorticoid production and dysregulated IGF-1 signaling altogether create an catabolic environment that accelerates activation of protein degradation, suppress protein synthesis, and impaired muscle regeneration (Wang et al., 2022). The role of exosomal miRNA derived from kidney in CKD on skeletal muscle disorders are far from clear. Nevertheless, an early study identified 12 miRNAs that are differentially expressed in skeletal muscle between normal and CKD mice (Wang et al., 2011), and among the differentially expressed miRNAs, miR-29 was significantly downregulated in skeletal muscle of CKD. The study showed that the inflammatory microenvironment activated NF-κB signaling that suppressed the level of miR29, and in turn inhibited the genes that promoted myogenic differentiation. By overexpressing miR29 in myoblasts from CKD muscle, myogenic differentiation was improved. Overexpression of miR-486, which was also reported downregulated in CKD muscle, exhibited protective effects by inhibiting muscle degradation in CKD mice. The above data suggested that downregulation of certain miRs accounted for the muscle wasting phenotypes in CKD.

Recent research shows that the crosstalk between skeletal muscle and the kidneys may retard the progression of CKD (Figure 1B). Evidence has showed that expressions of some exosomal miRNAs in CKD are sensitive to (and also response to) exercises. For examples, miR-1 and miR-206 were responsive to low frequency electrical stimulation that promoted myogenesis in CKD muscle (Chen et al., 2010; Hu et al., 2015). Several muscle-enriched miRNAs are secreted into general circulation. It is not surprising that muscular disorders showing altered levels of muscle-enriched or even muscle-specific miRNAs can exert their effects on distal target tissues. For the 5/6 nephrectomy model, the nephrectomized mice showed reduced expression of miR-23a in muscle compared with controls, whereas exercise increased the levels of miR-23a and miR-27a in the nephrectomy mice (Wang et al., 2017). Overexpression of precursor miR-23a and miR-27a may elevate the levels of mature miR-23a and miR-27a in circulating serum exosomes and attenuate muscle loss, reduce myostatin, and increase expression of markers of muscle regeneration (Wang et al., 2017). The same research group has reported in later study that miRNA-26a levels were reduced in both cardiac and skeletal muscles of 5/6 nephrectomy mice as compared with control mice (Wang et al., 2019b). An injection of Exo/miR-26a can prevent CKD-induced skeletal muscle wasting and attenuate cardiomyopathy in 5/6 nephrectomy mice via exosome-mediated miR-26a-regulated insulin resistance and FoxO1 (Wang et al., 2019b).

For the UUO model, Wang et al. reported that the miR-29a level was downregulated in both kidney and skeletal muscle of UUO mice (Wang et al., 2020). Injection of AAV-miR-29a into the tibialis anterior muscles not only inhibited YY1 and myostatin in skeletal muscles, but also suppressed fibrosis-related proteins (TGF-β1, TGF-β3, and collagen 1A1) in the kidney. After an injection of AAV-GFP into tibialis anterior muscles, the fluorescence levels of AAV-GFP were increased in the kidney and non-injected muscle observed under an *in vivo* Xtreme camera system. Interestingly, the kidneys showed the strongest fluorescence compared with other organs in UUO mice. The level of miR-29a was significantly higher in the obstructed kidney than in the unobstructed kidney of the UUO mice after injecting them with AAV-miR-29a, which means that injured organs may have a higher capacity to recruit exosomal miRNAs than uninjured organs (Wang et al., 2020). The investigators have created a gene-activated engineered exosome that specifically target organs expressing the acetylcholine receptor, such as the kidney (Wang et al., 2019a). The intervention of Exo/miR-29 increased the muscle’s cross-sectional area and ameliorated renal fibrosis in UUO mice. The authors confirmed that the decreased renal fibrosis after muscular injection of Exo/miR-29 was due to the increased circulation of exosome-encapsulated miR-29 (Wang et al., 2019a). In another study, intramuscular injection of exosomes with high miR-26 content prevented muscle atrophy by inhibiting FoxO1 and ameliorated renal fibrosis by suppressing connective tissue growth factor; they also demonstrated that exosomes originating at the muscle can target the kidney (Zhang et al., 2019).

For the diabetic nephropathy model, researchers also demonstrated the role of miRNAs in mediating crosstalk between skeletal muscle and the kidneys. Zhang et al. found that miR-23a/27a in muscle prevents diabetes-induced reduction of the muscle’s cross-sectional area and function and attenuates renal fibrosis lesions via muscle–kidney crosstalk (Zhang et al., 2018). This study supports the potential therapeutic applications of exosome delivery of miRNAs to prevent or treat sarcopenia and kidney injury in people with CKD.

## Summary and conclusion

In this review, we discussed the roles of exosomes and exosomal miRNAs in the skeletal muscle–kidney crosstalk in people with CKD. Accumulated evidence demonstrates that skeletal-muscle-derived exosomal miRNAs prevent the progression of CKD in different animal models. This phenomenon holds great potential for the development of strategies to treat complications arising from kidney diseases. Exosomes and exosomal miRNAs derived from the kidney may also interfere with skeletal muscle physiology and skeletal muscle disorders. Further studies are required to fully illustrate the signaling cascades of the two-way skeletal muscle–kidney crosstalk.
